# New Insight into Utilization of Fish By-Product Proteins and Their Skin Health Promoting Effects

**DOI:** 10.3390/md22050215

**Published:** 2024-05-09

**Authors:** Dongcheng Liu, Yongxin Ren, Saiyi Zhong, Baojun Xu

**Affiliations:** 1 Food Science and Technology Program, Department of Life Sciences, BNU-HKBU United International College, Zhuhai 519087, China; liudongcheng@uic.edu.cn (D.L.); 19927533501@163.com (Y.R.); 2 Guangdong Provincial Key Laboratory of Aquatic Product Processing and Safety, College of Food Science and Technology, Guangdong Ocean University, Zhanjiang 524088, China

**Keywords:** fish by-products, skin photoaging, fish protein, dermatology

## Abstract

In regions reliant on fisheries for livelihoods, a significant number of fish by-products are generated annually due to processing. These discarded parts contain valuable biological resources, such as proteins, fish oils, and trace elements, thus holding enormous potential for reutilization. In recent years, fish by-product proteins have been widely utilized in skincare products due to their rich collagen content, biosafety, and biocompatibility. This review summarizes the research into and applications of fish by-product proteins in skin health, including alleviating oxidative stress and skin inflammation, reducing DNA damage, mitigating melanin production, improving skin hydration, slowing skin matrix degradation, and promoting synthesis. Additionally, the possibility of improving skin health by improving the abundance of gut microbiota is also discussed. This review underscores the importance of fish by-product proteins in the fisheries, food processing, cosmetics, and biomedical industries.

## 1. Current Situation of Fish Production

The globally explosive population growth and the increasing expectation of high-quality food products have driven the evolution of the food manufacturing industry [1]. Due to the limited and overexploited natural sources on land, efforts to improve food research and development have shifted towards the aquatic environment, which covers 70.8% of the Earth’s surface. As a food source, aquatic products provide a wealth of nutrients, containing high-quality proteins, carbohydrates, vitamins, and minerals [2]. In 2020, global fisheries aquaculture production and first sale value reached 178 million tons and 406 billion US dollars, respectively [3].

Among various aquatic products, fish is believed to be a critical component of a healthy diet. Studies reported by Salindeho et al. [4] and Siscovick et al. [5] showed that fish products with high bioactive peptides have potential antioxidant, anti-inflammation, anticancer, and antimicrobial effects. Driven by modern techniques of capture fisheries and the rapid development of fishery aquaculture, fish consumption has become the dominant source of aquatic protein intake. Worldwide, fish consumption contributes approximately 20% of animal protein intake. This percentage rises above 50% for coastal and island developing countries, such as Bangladesh, Cambodia, and Ghana [6].

## 2. Profiles of Fish by-Products

After harvest, almost all fish in the fishery industry undergo further processing before being sent to the market [7]. Depending on the species and size of fish, more than 45% of fish biomass consists of inedible parts, including fish scales, skin, fins, head, bones, and internal organs [8]. Therefore, fish processing is crucial for the fishery business because it reduces the transportation cost of inedible parts and ensures the consistency and safety of products. Removing parts, such as fish fins, can reduce the likelihood of fish bones puncturing the sealed packaging during transport. Similarly, removing viscus, which contains bacteria and enzymes, can help to mitigate the risk of infection and protein–lipid hydrolysis [9]. The removed fish by-products have significant potential to provide various health benefits to humans. Studies have shown that daily supplementation of fish scales can promote the synthesis of chitin and chitosan, which possess various health benefits, such as their antihypertensive, antihyperlipidemic, and immunomodulatory properties [10,11].

## 3. Utilization of Fish By-Products

Fish by-products are prone to spoilage when exposed to air because they are typically rich in moisture and nutrient contents. In most situations, these by-products are discarded due to inadequate techniques and resources for effective utilization, even though they hold potential value as sources of nutrients and bioactive compounds [12]. For example, the flesh of oily fish and the liver of lean fish are rich in omega-3 and omega-6 polyunsaturated fatty acids, which have functional effects on human health [13]. After harvest, if these fatty acids are not extracted and refrigerated immediately, they become vulnerable to oxidation, quickly leading to rancidity. Hitherto, fish by-products are fractionally used for manufacturing low-profitability products, such as feed supplements, fertilizer, fishmeal, and fish oil [14,15]. In light of shipping costs, some fish by-products are either utilized in aquaculture feed, leading to ethical problems, or disposed of in landfills, contributing to environmental concerns. Consequently, the comprehensive implementation of such resources is gaining increasing prominence. Notably, global demand for fish as a good source of protein is rising annually. However, the proportion of fish that are captured in the wild is gradually declining. Therefore, the majority of the growth is attributed to aquaculture. In 2018, aquaculture contributed 46% of global fish production, almost doubling from 25.7% in 2000 [16]. Due to the increasing proportion of fish being harvested from aquaculture, the uniformity of fish species and size is guaranteed, allowing for standardized treatment of fish by-products and subsequent development of a standard operating procedure (SOP) in large-scale production. Many researchers have recently focused on developing methods to utilize fish by-products to produce healthy goods, potentially contributing to economic growth and sustainable development.

## 4. Health Benefits of Fish By-Product Proteins

Fish is a highly nutritious food source, with fish flesh containing a significant number of high-quality proteins. Therefore, many studies have d the healthy effect of use of fish proteins by consuming fish meat [17,18]. Fish by-products, including fish bones, skin, and scales, are also valuable sources of fish proteins and have been found to contain all the essential amino acids, including sulfur-containing amino acids that are typically absent in plant-based proteins [19]. Fish bone has a similar structure to mammalian bone, possessing osteocytes and osteoclasts but lacking osteons [20]. Osteonectin and calcitonin, proteins found in fish bone, play roles in modulating osteogenesis and osteoblastic differentiation, respectively. They function similarly to their mammalian counterparts in these biological processes [21]. Fish skin and scales contain a high collagen content, a structural protein that provides high tensile strength and stability to complex functions. In addition to collagen, certain non-collagenous proteins, such as elastin and casein, have been discovered in fish skin. Consuming these fish by-product proteins can provide various health benefits to the human body. For instance, fish collagen is known to aid in maintaining healthy skin, joints, bones, and blood vessels [22]. Based on Halim et al. [23], the non-collagenous bioactive proteins of fish have enabled the development of functional foods with potential benefits, such as their anti-oxidative, anti-hypertensive, and anti-proliferative properties. Fish collagen is widely used to replace terrestrial source collagen due to the lower risk of diseases, such as mad cow disease from bovine collagen, pseudo-rabies from porcine collagen, and avian flu from poultry sources [24]. Apart from these, the usage of fish collagen may be less restricted by religious elements. For example, Hindus cannot use cow products, and Muslims should not use porcine products and non-Halal certified products, while fish collagen does not have these limitations. Proteins synthesized by trans-gene microbes have recently been applied as a potential alternative source. Nevertheless, their bioactivity, stability, and safety are not comparable with fish by-product proteins [25,26].

## 5. Pre-treatment of Fish By-Products

Fish by-products are difficult to consume directly as food due to their unpleasant mouthfeel, complex texture, and a strong fishy odor that persists even after thorough washing. Therefore, an extraction technique is necessary to isolate the bioactive components from fish by-products.

The protein extraction procedure from fish by-products comprises three major steps: pre-treatment, extraction, and recovery. Initially, the objective of pre-treatment is to remove non-protein substances and, consequently, increase extraction efficacy [27]. The typical pre-treatment technique involves the use of butyl alcohol to remove organic substances, such as fats and pigments, from fish head and skin [28]. Butyl alcohol is a lipophilic and non-polarity solvent, which exhibits an affinity for lipids but repels protein by not forming polar bonds. Minerals are undesired material in fish by-products. They can maintain the structure of fish frames and scales and bind the fish protein within those by-products. During extraction, these minerals can interact with amino acid residues in the protein, forming salt bridges or ionic bonds [29]. This interaction has an adverse impact on the solubility and quality of the protein extract. Consequently, it is necessary to eliminate the mineral contents in raw materials. Calcium is a typical mineral in fish heads, bones, and scales. Commercially, the removal of minerals from fish by-products is commonly achieved using low concentrations of hydrochloric acid, citric acid, or ethylenediaminetetraacetic acid (EDTA) [30]. However, using acid to remove minerals can cause protein loss in the process. The solubility of protein is increased in a low-pH environment. Therefore, controlling the concentration and duration of acid treatment is crucial. In most cases, collagen in fish by-products is the target for protein extraction. A low concentration of sodium hydroxide is applied to dissolve non-collagenous protein by forming soluble salt. It can remove unwanted components and induce swelling in the sample matrix by increasing the transfer rate that facilitates collagen extraction [31,32].

## 6. Extraction of Fish By-Product Proteins

Acid extraction is a widely adopted method for protein isolation. In the 1960s, Bailey revealed that protein solubility is closely related to the destruction of ionic bonds and Schiff-base. Using acid extraction can provide a low ion concentration and acid environment, which is detrimental to those intermolecular connections. Acid extraction is the most preferred method for protein extraction. The current research interest is to compare extraction efficiencies of various acid solutions, including acetic acid, citric acid, hydrochloric acid, lactic acid, sulfuric acid, and formic acid [33,34]. The extraction procedure typically takes 12 to 48 h for optimal matrix utilization, depending on the acid solution concentration. The extraction process needs to be carried out at low temperatures to ensure the quality of protein extraction for biomedical applications. The study by Bhuimbar and Bhagwat [33] used different acids to extract collagen from catfish skin. The highest yield rate was 45% by lactic acid. The recovery was accomplished through salting out or precipitation and was further refined by dialysis in pure water to remove the salt.

Typically, fish collagen cannot be fully dissolved in an acidic medium. Certain collagens may be enclosed within the raw materials and prove challenging to extract. In this case, enzymes, such as pepsin, trypsin, and papain, can be added to facilitate the extraction rate [35]. A study indicated that enzyme extraction has no influence on the triple-helix structure of collagen [36]. Pepsin is the preferred choice among all enzymes because the optimal pH for pepsin is 2.0–3.0, which is suitable for an acid environment. Collagen extracted using acid alone is commonly known as acid-soluble collagen (ASC), while collagen extracted using a combination of acid and enzymatic treatment is referred to as enzyme-soluble collagen (ESC), where the specific enzyme used may vary. Enzyme-assisted acid extraction is a promising method for collagen extraction as it enhances extraction efficacy and facilitates the release of the bioactive components of collagen.

In both commercial application and research investigation, fish proteins are commonly digested into peptides [37]. The fish peptides have smaller molecular weight and higher bioactivity compared with proteins. Therefore, the bioavailability of those peptides is much higher than that of proteins in in vivo experiments [38]. The typical method is enzymatic hydrolysis, including pepsin, papain, alkaline, neutral, and acid protease utilization [37,39,40]. The difference between various enzymatic treatments for fish proteins is that they work on different protein bonds, producing peptides with different amino acid sequences and spatial structures, which becomes a research interest for investigation of the connection between peptide characteristics and its bioactivity. In addition, to receive a peptide with lower molecular weight, researchers used multiple enzymes to hydrolyze the fish by-product proteins [40,41]. Robert et al. (2015) reported that they hydrolyzed the tilapia skin and scale proteins by a bacillus protease complex with one-time hydrolysis. They identified over 1374 oligopeptides and polypeptides from the hydrolysis products and found that the hydrolysates exhibit antimicrobial activity [41].

## 7. Skin Health Promotion Effects of Fish By-Product Proteins

**Figure 1:**
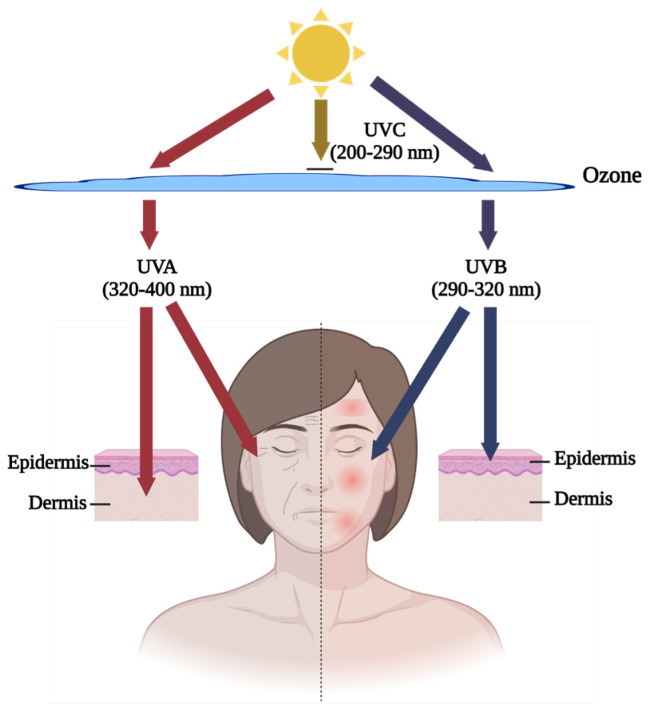
The effects of UV radiation on various layers of the skin. Graphical elements applied were from BioRender (https://www.biorender.com, accessed on 28 March 2024).

Skin aging, caused by intrinsic and extrinsic factors, is one problem that could be ameliorated by fish by-product proteins. Intrinsic factors are raised by the natural deterioration of human skin. This process occurs spontaneously with time and contributes to 20% of skin aging [42]. Extrinsic factors account for 80% of skin aging and have various sources, including sunlight radiation, air pollution, smoking, and hazardous chemicals [43]. Ultra-violet (UV) exposure is the most common hazard source of extrinsic factors, also called skin photoaging [44]. Figure 1 below shows that UVB (290–320 nm) has a weak penetration ability and causes harmful effects on the epidermis, such as inflammatory symptoms, like redness, swelling, and inflammation, on the surface of the skin, while UVA (320–400 nm) can penetrate into the dermis, a deeper layer of skin, and cause superficial dullness and deep wrinkles [45]. In the research field, the well-recognized in vitro models for human skin epidermis and dermis are human keratinocytes (HaCaT) and human dermal fibroblasts (HDF), respectively [46,47,48]. Due to the low survival rate and slow proliferation of HDF, some studies have utilized mouse fibroblast L929 cells as an in vitro model [49]. For in vivo study, the fur of regular mice/rats can block UV irradiation against the skin. The ideal in vivo model is that of hairless mice [50,51,52] (Table 1). Some researchers suggest using sodium sulfide or physical shaving to remove normal mice hair [53,54]. However, the chemical method might cause skin redness or allergy reactions due to its corrosiveness, similar to the photoaging effect, and cause disruption to observation. The shaving method is feasible but has the risk of damaging skin integrity. Therefore, hairless mice are still the optimal option for the in vivo model.

Fish by-product proteins have been proven to be effective in clinical research. Fish collagen is applied in some cosmetic and skin health products, and consumers are convinced that these products can be a collagen supplement for protecting against skin collagen decline. Nile tilapia is known for its fast reproduction rate and high global production. As shown in Table 1, the protein derived from Nile tilapia by-products is frequently investigated for its anti-photoaging properties. However, due to concerns about biosafety, protein quality, and product diversification, more studies have focused on exploring the potential of by-product proteins from other fish species with anti-photoaging effects, as shown in Table 2. Maia Campos et al. [57] conducted a double-blind, placebo-controlled clinical study on 46 subjects of oral supplement hydrolyzed fish cartilage. The results indicated that, compared with the placebo group, the fish cartilage supplement group had a significant reduction in wrinkles and an increase in dermis echogenicity, improving the structure of the skin. Based on reflectance confocal microscopy, oral supplements of fish by-product proteins can also improve skin collagen morphology and prevent elastosis. The human efficacy experiments provided strong evidence that collagen replenishment can significantly improve skin structure and quality.

### 7.1. Fish By-Product Proteins for Skin Oxidative Stress Relief

**Figure 2:**
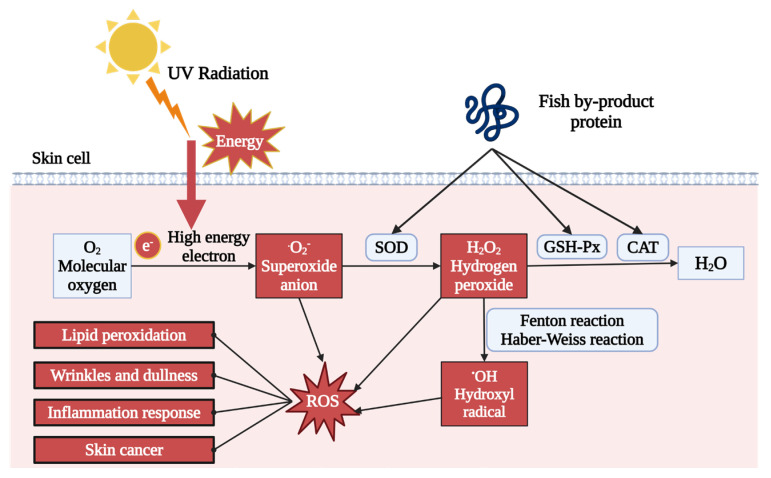
Fish by-product proteins alleviate oxidative stress in skin. Graphical elements applied were from BioRender (https://www.biorender.com, accessed on 28 March 2024).

One of the primary outcomes of UV exposure is the production of excessive reactive oxygen species (ROS), causing oxidative stress in the dermal layer. UV irradiation creates high-energy electrons, which can combine with oxygen to generate free radicals, such as superoxide anion (O_2_^−^), hydrogen peroxide (H_2_O_2_), and hydroxyl radical (OH). The accumulation of those free radicals induces oxidative stress. As described in Table 1 and Table 2, both in vivo and in vitro treatment of fish by-product proteins can significantly reduce the ROS level detected by the 2′,7′-dichlorodihydrofluorescein diacetate (DCFH-DA) fluorescent probe [49,60,61]. As shown in Figure 2, other supporting evidence indicated that antioxidant enzyme activity was also upregulated by fish by-product protein supplement, including superoxide dismutase (SOD), glutathione peroxidase (GSH-Px), and catalase (CAT) [50,55,59]. The SOD could accelerate the decomposition of superoxide anion with high toxicity into less toxic chemical hydrogen peroxide. GSH-Px and CAT further catalyzed the hydrogen peroxide into non-toxic alcohol and water. The raising of those enzymes can protect dermal cells from oxidative stress by eliminating and neutralizing ROS, thereby maintaining the redox balance. Other ROS relative photoaging effect can also be regulated by fish by-product proteins, such as lipid peroxidation indicator malondialdehyde (MDA) and mitochondrial membrane potential (MMP) [52,59].

### 7.2. Melanogenesis Prevention by Fish By-Product Proteins

**Figure 3:**
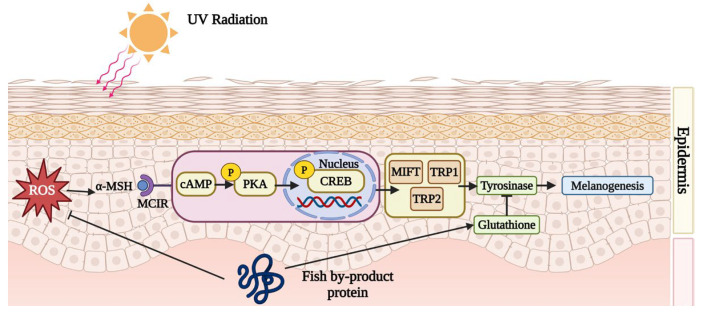
Fish by-product proteins downregulate melanogenesis. Graphical elements applied were from BioRender (https://www.biorender.com, accessed on 28 March 2024).

Fish by-product protein supplements can inhibit melanin synthesis. When UV irradiates the skin, the dermis activates melanogenesis and synthesizes lots of melanin to protect vulnerable skin. However, the accumulation of melanin might induce skin darkness, dullness, and freckles. Fish by-product proteins can retard the melanin synthesis cyclic adenosine 3,5-monophosphate (cAMP) pathway to improve skin condition. According to Figure 3, after UV exposure, the dermis would secret an endocrine hormone, α-melanocyte-stimulating hormone (α-MSH), to combine with melanocortin-1 receptor (MC1R). In the study conducted by Lee et al. (2022), it was found that collagen peptide, originating from tilapia (*Oreochromis niloticus*) skin, can down-regulate the production of excessive α-MSH [56]. The interaction between α-MSH and MC1R triggers a cascade of cytokines, including cAMP, p-PKA, p-CREB, MITF, TRP1, and TRP2, which would up-regulate the protein expression of tyrosinase. More tyrosine would be catalyzed by tyrosinase into catechol and eventually into melanin. By inhibiting the cAMP/α-MSH/MC1R pathway, melanin synthesis can be effectively suppressed. The proteins from the skin of Nile tilapia (*Oreochromis niloticus*) protect against melanogenesis by inhibiting the cAMP pathway and tyrosinase activity [56]. In addition, Li et al. (2022) demonstrated that protein from the scale of silver carp (*Hypophthalmichthys molitrix*) helps to elevate the levels of glutathione (GSH), which serves as an antagonist to tyrosinase [62].

### 7.3. Improvement of Skin Hydration by Fish By-Product Proteins

Fish by-product protein supplements can improve the moisture situation in the skin, which is achieved in two significant ways. Firstly, it can stimulate hyaluronic acid synthesis, which can boost skin moisture retention [50,56]. Secondly, by promoting sphingomyelin production, the water loss in the skin can be reduced [51]. Cho et al. (2023) found that oral supplementation with collagen peptides from tilapia and pangasius skin can promote hyaluronic acid synthesis in the skin [52]. This can increase the expression of hyaluronan synthase HAS1 and HAS2, thereby enhancing the catalysis of UDP-N-acetylglucosamine and UDP-glucuronic acid (UDP-GlcNAc) to synthesize the core chain of hyaluronic acid. It can also downregulate the expression of hyaluronidase 2, thereby decreasing the degradation of hyaluronic acid [52]. Skin moisturization can enhance the enzyme activity of DEGS1, leading to increased sphingomyelin production in cells, thereby maintaining the skin barrier function and preventing water loss.

### 7.4. Influence of Fish By-Product Proteins on Photoaging-Induced DNA Damage

**Figure 4:**
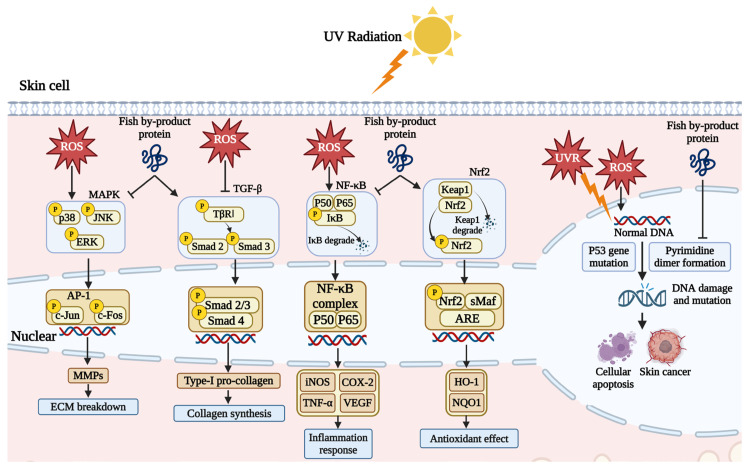
Fish by-product proteins mediate signaling pathways and DNA damage. Graphical elements applied were from BioRender (https://www.biorender.com, accessed on 28 March 2024).

Fish by-product proteins can repair the chromosome damage caused by UV exposure. High-energy UV exposure can trigger the pyrimidine dimer formation of the single DNA strand, where the cytosine (C) and thymine (T) are easily combined, blocking the transcription and replication process. This phenomenon can result in DNA strand breakage and base mismatching [63]. As shown in Figure 4, UV exposure can cause p53 gene mutation and halt the self-repairment of DNA strands, resulting in pre-malignant skin lesions and skin cancer [64]. Chen et al. (2018) found that collagen from the scale of milkfish (*Chanos chanos*) can help to protect the skin DNA. The agarose gel electrophoresis result showed that UV exposure can destroy supercoil O-form and S-form DNA in pUC119 plasmid and synthesized L-form. Supplement with fish collagen can prevent this degradation [61]. Chen et al., (2022) used protein from the skin of large hybrid sturgeon (*Huso dauricus × Acipenser schrenckii*) to rescue UVB-damaged skin cells. Hoechst 33342 staining results revealed that fish protein reduced DNA condensation in a dose-dependent manner [49].

### 7.5. Impact on Skin Extracellular Matrix by Fish By-Product Proteins

Fish by-product proteins can alter the extracellular matrix (ECM) content and improve skin condition by regulating two signaling pathways. As illustrated in Figure 4, skin cells absorb a lot of energy and generate a significant amount of ROS after exposure to ultraviolet radiation. In skin cells, ROS can activate the mitogen-activated protein kinase (MAPK) pathway, leading to the production of matrix metalloproteinases (MMPs), which degrade the ECM, cause dermal atrophy, and disrupt of structural integrity, resulting in loss of skin elasticity and sagging [65]. Numerous experiments have shown that supplementation with fish by-product proteins significantly inhibits the activation of the MAPK signaling pathway [49,50,55,56]. Fish by-product proteins reduce the phosphorylation expression of p38, JNK, and ERK, decreasing the expression of downstream genes c-Jun and c-Fos. The transcription factor c-Jun is an important component of the transcriptional activation protein (AP-1), and its entry into the cell nucleus can promote the expression of MMPs. Therefore, inhibiting the expression of c-Jun and c-Fos can effectively intervene in skin photoaging by reducing the expression of MMPs.

Moreover, fish by-product proteins can activate the transforming growth factor-beta (TGF-β) signaling pathway to synthesize pro-collagen type Ⅰ, promoting the proliferation of fibroblasts and the synthesis of ECM components, such as collagen and elastin, which contribute to the skin repair and reconstruction process [66]. Collagen from the skin and scale of Nile tilapia (*O. niloticus*) has been shown to promote the expression of the TGF-β receptor and phosphorylation of Smad 2 and Smad 3 in both in vitro and in vivo studies [50,56]. Those cytokines combine and translocate into the cell nucleus, promoting pro-collagen expression [55]. Besides, the expression of Smad 7 can be inhibited, the over expression of which blocks the translocation process.

### 7.6. Fish By-Product Proteins for Photoaging-Induced Skin Inflammatory

Fish by-product proteins have been found to regulate inflammatory responses in the skin. The nuclear factor kappa B (NF-κB) signaling pathway closely associated with ROS is a critical mediating factor in the process of skin inflammation and aging. Inhibition of inflammation is very important in the anti-photoaging effect. Much research has shown that P50, P65, and IκB are important cytokines to mediate inflammatory response. As indicated in Figure 4, under normal conditions, P50, P65, and IκB kinase form a stable complex in the cytoplasm. The oxidative stress from UV exposure stimulates the phosphorylation of IκB and causes IκB degradation. Subsequently, the remaining P50 and P65 heterodimers translocate to the nucleus [67]. The expression and secretion of cytokines, including TNF-α, COX-2, VEGF, and iNOS, could induce photoaging-associated inflammation. Tumor necrosis factor-α (TNF-α) is a pleiotropic cytokine mediating inflammation response. It can promote skin cell necrosis and apoptosis, increase the expression of MMPs, degrade the ECM components, and cause photoaging symptoms, like skin relaxation, wrinkles, and erythema [68]. The over-expression of cyclooxygenase-2 (COX-2) stimulates the secretion of prostaglandin E2 (PGE2), which mediates the cell life cycle and induces cell invasiveness and oncogenicity in the worst case [69]. In comparison, vascular endothelial growth factor (VEGF) is a relatively moderate cytokine. In the skin cell, thrombospondin-1 (TSP-1) and VEGF have conjugate antagonistic effects against each other. TSP-1 can inhibit endothelial cell proliferation and migration, reducing angiogenesis potently [70]. Acute UVR strongly upregulates VEGF expression, and the TSP-1/VEGF ratio decreases dramatically. The equilibrium disruption shifts the status from vascular quiescence to a pro-angiogenic state, leading to early skin senescence characterized as ECM degradation [71]. Nitric oxide (NO) is an easily detectable inflammatory cytokine produced by inducible nitric oxide synthase (iNOS) in skin cells under UV irradiation. Recent experiments have shown that fish bone and skin protein can reduce the phosphorylation of IκB, the levels of P65, and downstream regulatory proteins IL-1β, IL-6, TNF-α, COX-2, and iNOS [56,60]. In addition to downregulating the expression of signaling pathways involved in inflammation, fish by-product proteins can reduce oxidative stress in skin cells by activating the nuclear factor erythroid 2-related factor 2 (Nrf-2). Based on the study of Kong et al. (2023), the activation of the Nrf-2 signaling pathway was observed in UV-irradiated HaCaT cells following treatment with cardiac arterial proteins from skipjack tuna [59]. This promoted the dissociation of Nrf2 from Keap1, allowing its nuclear translocation and binding to antioxidant response elements (ARE). Consequently, the effector proteins heme oxygenase-1 (HO-1) and NAD(P)H quinone-oxidoreductase-1 (NQO1) expression was enhanced, facilitating the redox balance in the skin.

### 7.7. Gut Microbiota Balance and Fish By-Product Proteins

The relationship between gut microbiota and skin health is closely connected and mutually influential. The concept of the “gut–skin axis” exists, where the gut microbiota can transmit signaling molecules to immune cells in skin through interactions between the gut and skin. Therefore, the dysbiosis of the gut microbial community can substantially impact the overall well-being of the skin. Numerous studies have provided evidence that the gut microbiota plays a critical role in regulating skin photoaging-associated conditions, including inflammatory response and barrier function [72,73,74]. Specifically, gut microbiota can regulate the production and secretion of immune active components (such as cytokines and immunoglobulins), affecting the degree and duration of skin inflammatory reactions. The study by Geng et al. in 2024 found a high positive or negative correlation between UV-induced skin photoaging and changes in the abundance of specific strains, showing both positive and negative associations [75]. By comparing the changes in the abundance of the top 30 gut microbiota strains at the genus level before and after UV exposure in mice, they concluded that *Mucispirillum*, *Bifidobacterium*, *Parasutterella*, and *Dubosiella* were correlated with skin health indicators, like epidermal thickness, hydration, wrinkle volume, HA content, and collagen density, suggesting a relationship between these strains and skin photoaging. The result allows for regulating and maintaining skin health status by adjusting the balance of the gut microbiota. Studies have shown that altering the composition of the gut microbiota through fecal transplantation significantly improved the damaged state of skin photoaging [76]. This was mainly achieved by reducing the TNFR1/TNFR2-mediated MAPK pathway expression and altering intestinal amino acid metabolism [74]. Previous studies found that the metabolites of fish by-product proteins can alter the abundance of gut microbiota by changing the gut substrate and environment [77]. In addition, a high protein diet can also alter the abundance of gut microbiota to increase the gut level of short-chain fatty acids (SCFAs), which play a crucial role in enhancing the health of intestinal epithelial cells by promoting anti-inflammatory and immune-regulatory functions [78]. The gut–skin axis further explains how SCFAs impact skin health, highlighting the intricate connection between the gut microbiome and skin physiology. Although existent research has not established a direct correlation between the enhancement of gut microbiota from fish by-product proteins intake and the imbalance resulting from photoaging, the relationship between the two is notably intertwined. Therefore, the connection between them is worth further exploration.

## 8. Conclusions

With the advancement of science and technology, biological resources are being fully utilized. Especially for coastal areas, with the continuous maturation of aquaculture technologies, in-depth research on the effective utilization of fish by-products has followed. This article discusses the current extraction of fish by-product proteins and their application in anti-photoaging. It has been proven by many mechanism studies that fish by-product proteins can relax the skin photoaging effect. Free radical synthesis, along with the following extracellular matrix breakdown and inflammatory reaction, can be downregulated by fish by-product proteins. Fish by-product protein treatments can suppress DNA damage and melanin synthesis from UV radiation. In addition, skin moisture can be improved by fish by-product protein supplements via increasing hyaluronic acid content and forming a sphingomyelin shell.

## Figures and Tables

**Table 1:** Nile tilapia by-product proteins against skin photoaging.

Source	Model	Molecular Targets	Biological Activity	References
Scale of Nile tilapia (*O. niloticus*)	UVB-induced HaCaT UVB-induced HS27 UVB-induced SKH-1 mice	↑: SOD, CAT, GSH-Px	↑: Antioxidant capacity	[50]
		↑: *TGF-βRI*, Smad3, *Collagen I*, *Pro-collagen I*	↑: ECM synthesis	
		↑: *HAS (1–3)*, *LCB1(SPT)*, *Elastin*, *Fibrillin-1*, Hyaluronic acid	↑: Skin hydration	
		↑: CerS4, *DEGS1*, Sphingomyelin	↓: Moisture loss	
		↓: JNK, c-Fos, c-Jun, MMP-(1,2,9)	↓: ECM breakdown	
		↓: IκB, P65, COX-2, TNF-α, IL-1β, IL-6	↓: Inflammation	
Scale of Nile tilapia (*O. niloticus*)	UVB-induced HaCaT UVB-induced SKH-1 mice	↑: *LCB1*, *GLcNAc*, *UGTrel7*, *Elastin*, HAS2, Hyaluronic acid	↑: Skin hydration	[51]
		↑: CerS4, *DEGS1*, Sphingomyelin	↓: Moisture loss	
Skin of pangasius (*P. bocourti*) and tilapia (*O. niloticus*)	UVB-induced HaCaT UVB-induced HDF UVB-induced SKH-1 mice	↑: HAS2; *COL1A1*, HA; ↓: HYAL2, Elastase	↑: Skin hydration	[52]
		↑: *SOD*, *GSH-Px*, MDA; ↓: ROS	↑: Antioxidant capacity	
		↓: ERK, p38, JNK, MEK1/2,3,4, MMP-1,13	↓: ECM breakdown	
Scale of Nile tilapia (*O. niloticus*)	UVB-induced Hs27 IBMX-induced B16F10 UVB-induced SKH-1 mice	↑: GSH; ↓: PKA, cAMP, CREB, MITF, TRP-1, TRP-2, Melanin	↓: Melanogenesis	[55]
		↑: SOD, GSH-Px, CAT	↑: Antioxidant capacity	
		↑: *TGF-βRI*, Smad3, *Pro-collagen I*, *Collagen I*	↑: ECM synthesis	
		↓: JNK, c-Fos, c-Jun, MMP-1,3,9	↓: ECM breakdown	
		↓: IL-1β, IL-6, TNF-α, NO	↓: Inflammation	
Skin of Nile tilapia (*O. niloticus*)	UVB-induced HaCaT UVB-induced Hs27 IBMX-induced B16F10 UVB-induced SKH-1 mice	↑: *HAS1–3*, *LCB1(SPT)*, *DEGS1*, *Fibrillin-1*, *CerS4*, Hyaluronic acid, Sphingomyelin	↑: Skin hydration	[56]
		↑: GSH; ↓: cAMP, MITF, TRP-1,2, PKA, CREB	↓: Melanogenesis	
		↑: SOD, CAT, GSH-Px	↑: Antioxidant capacity	
		↑: *TGF-βRI*, Smad3, *Pro-collagen I*, *Collagen I*	↑: ECM synthesis	
		↓: JNK, c-Fos, c-Jun, MMP-1,3,9	↓: ECM breakdown	
		↓: Iκ-B, P65, COX-2, IL-1β, IL-6, TNF-α	↓: Inflammation	

**Table 2:** Other fish by-product proteins against skin photoaging.

Source	Model	Molecular Targets	Biological Activity	References
Cardiac arterial bulbs of skipjack tuna (*K. pelamis*)	UVB-induced HaCaT	↑: SOD, CAT, GSH-Px, MDA, Mitochondrial membrane potential; ↓: ROS	↑: Antioxidant capacity	[58,59]
		↑: Nrf2, HO-1, NQO1, Bcl-2. ↓: Cleaved-caspase-3,8,9, Bax	↑: Antioxidant capacity	
		↓: Hoechst 33342 Staining	↓: DNA damage	
Bone of silver carp (*H. molitrix*)	UVB-induced HaCaT UVB-induced L929 Human melanoma	↓: ROS, MDA	↑: Antioxidant capacity	[60]
		↓: TNF-α, IL-1β	↓: Inflammation	
		↓: Tyrosinase Activity, Melanin	↓: Melanogenesis	
Skin of large hybrid sturgeon (*H. Dauricus)*	UVB-induced L929 UVB-induced Zebrafish Embryo	↑: Pro-collagen I	↑: ECM synthesis	[49]
		↓: ROS, MDA,	↑: Antioxidant capacity	
		↓: *IL-1β, IL-6, TNF-α, Cox-2*	↑: Antioxidant capacity	
		↓: p38, Erk1/2, Jnk1/2/3, c-Jun, MMP-1,2,3	↓: ECM breakdown	
		↓: Hoechst 33342 Staining	↓: DNA damage	
Scale of milkfish (*C. Chanos*)	UV-induced HaCaT UV-induced plasmid	↑: O-form, S-form plasmid	↓: DNA damage	[61]
		↓: iNOS, NO	↓: Inflammation	
		↓: DPPH, ABTS, ROS	↑: Antioxidant capacity	
